# The identification of type I MADS box genes as the upstream activators of an endosperm-specific invertase inhibitor in Arabidopsis

**DOI:** 10.1186/s12870-021-03399-3

**Published:** 2022-01-06

**Authors:** Tobias Hoffmann, Xiuling Shi, Chuan-Yu Hsu, Aakilah Brown, Quintera Knight, La’ Shyra Courtney, Ruqiyah J. Mukarram, Dongfang Wang

**Affiliations:** 1 Biology Department, Spelman College, Atlanta, GA USA; 2 Institute for Genomics, Biocomputing and Biotechnology, Mississippi State University, Mississippi State, MS USA

**Keywords:** MADS-box, Invertase inhibitor, Endosperm, PRC2, Seed development

## Abstract

**Background:**

Nuclear endosperm development is a common mechanism among Angiosperms, including Arabidopsis. During nuclear development, the endosperm nuclei divide rapidly after fertilization without cytokinesis to enter the syncytial phase, which is then followed by the cellularized phase. The endosperm can be divided into three spatial domains with distinct functions: the micropylar, peripheral, and chalazal domains. Previously, we identified two putative small invertase inhibitors, InvINH1 and InvINH2, that are specifically expressed in the micropylar region of the syncytial endosperm. In addition, ectopically expressing InvINH1 in the cellularized endosperm led to a reduction in embryo growth rate. However, it is not clear what are the upstream regulators responsible for the specific expression of InvINHs in the syncytial endosperm.

**Results:**

Using protoplast transient expression system, we discovered that a group of type I MADS box transcription factors can form dimers to activate InvINH1 promoter. Promoter deletion assays carried out in the protoplast system revealed the presence of an enhancer region in InvINH1 promoter, which contains several consensus cis-elements for the MADS box proteins. Using promoter deletion assay *in planta*, we further demonstrated that this enhancer region is required for InvINH1 expression in the syncytial endosperm. One of the MADS box genes, AGL62, is a key transcription factor required for syncytial endosperm development. Using promoter-GFP reporter assay, we demonstrated that InvINH1 and InvINH2 are not expressed in *agl62* mutant seeds. Collectively, our data supports the role of AGL62 and other type I MADS box genes as the upstream activators of InvINHs expression in the syncytial endosperm.

**Conclusions:**

Our findings revealed several type I MADS box genes that are responsible for activating InvINH1 in the syncytial endosperm, which in turn regulates embryo growth rate during early stage of seed development.

**Supplementary Information:**

The online version contains supplementary material available at 10.1186/s12870-021-03399-3.

## Background

The seeds of angiosperms are made up of three distinct tissues, the seed coat, the embryo, and the endosperm. The embryo and the endosperm are both products of double fertilization [1, 2]. Besides supplying nutrients to support the growth of the embryo or the germinating seedling [3], the endosperm also influences the development of the neighboring embryo and seed coat [4–6]. Similar to most angiosperms, Arabidopsis has a nuclear endosperm that starts with a syncytial phase marked by rapid nuclear division without cytokinesis [3, 7]. The nuclear endosperm further differentiates to form three distinct domains, the micropylar domain surrounding the embryo, the chalazal domain next to the maternal vasculature, and the peripheral domain in the center [3]. After cellularization, the endosperm either persists as the main storage tissue in the seed, or is gradually absorbed by the growing embryo [3, 7].

Several regulators have been reported to control the developmental transition from syncytial endosperm to cellularized endosperm. The syncytial program is negatively regulated by a chromatin repressive complex, the FIS-PRC2 (Polycomb Repressive Complex 2) complex. In *fis* mutants such as *mea*, *fis2*, *fie*, and *msi1*, the endosperm fails to cellularize, which in turn leads to embryo abortion [8–11]. The PRC2 complex carries histone H3 lysine 27 methyltransferase activity and is known to regulate many important developmental transitions in both plants and animals [12–15]. Besides the FIS-PRC2 complex, the timing of endosperm cellularization is also sensitive to global DNA methylation level and parental genome dosage [16–19], indicating that large-scale chromatin remodeling events occur during endosperm cellularization. Interestingly, the syncytial program is positively regulated by a type I MADS box transcription factor, AGL62, which is a direct downstream target of the FIS-PRC2 complex [20]. In *agl62* mutants, the endosperm enters cellularization prematurely [21]. Therefore, the transition from syncytial to cellularized endosperm is likely achieved through the FIS-PRC2-mediated suppression of AGL62.

Even though regulators specifically expressed in the micropylar or the chalazal endosperm have been reported [6, 22–24], very few regulators have been shown to control how endosperm differentiates to form the three distinct spatial domains. AGL62 and its homologs have emerged as the potential regulators of syncytial endosperm development, since a large number of the type I MADS box genes are specifically expressed in the syncytial endosperm, many of which are in a domain-specific manner [25, 26]. In addition, yeast two-hybrid data suggested that these type I MADS box proteins form a network of protein dimers centered around AGL62 [26, 27]. The consensus motif bound by type I MADS-box proteins has not been studies as extensively as the type II MADS-box proteins, which bind a consensus sequence CC(A/T)_6_GG called the CArG-box [28, 29]. The type II MADS-box proteins are often recruited by multiple CArG motifs to the target promoter to form higher-order heterotetrameric complexes, many of which are regulators of floral organ identity [30, 31]. Motifs similar to the CArG-box were also reported as the binding site for one of the type I MADS-box genes, PHE1/AGL37 [32].

Prior work in our lab has identified a putative invertase inhibitor, InvINH1, as a suppressor of embryo growth [33]. InvINH1 is preferentially expressed in the micropylar endosperm during the syncytial phase. In *fis2* mutant, InvINH1 mRNA level is dramatically up-regulated [33]. Our lab also contributed to the discovery of a subset of type I MADS-box genes, termed the C2 AGLs, that are expressed in the syncytial endosperm and up-regulated in FIS-PRC2 mutant [26]. The overlapping expression pattern of InvINH1 and C2 AGLs prompted us to investigate whether C2 AGLs could regulate the expression of InvINH1. Our data indicated that several C2 AGLs formed dimers to activate InvINH1 expression. Moreover, the InvINH1 promoter contains an enhancer region enriched with CArG motifs.

## Results

### AGL dimers activate InvINH1 promoter in protoplast transient assay

Our prior work has shown that invertase inhibitor 1 (InvINH1) is expressed in the syncytial endosperm and is up-regulated in PRC2 mutants such as *fis2* [8]. To identify the upstream regulators of InvINH1, we first examined transcription factors that are known to share similar expression patterns as InvINH1, such as type I MADS-box genes. The type I MADS-box transcription factors of the AGAMOUS-LIKE (AGL) family are enriched with genes expressed in the syncytial endosperm [25, 26]. Moreover, 16 of the type I MADS-box genes, termed C2 AGLs, are also up-regulated in PRC2 mutants [26]. Because C2 AGLs and InvINH1 share similar spatial and temporal expression pattern, we carried out a series of transient expression assays to determine if C2 AGLs could directly regulate the expression of the full-length InvINH1 promoter-GUS reporter (pInvINH1-GUS) in isolated Arabidopsis leaf mesophyll protoplasts.

**Fig. 1:**
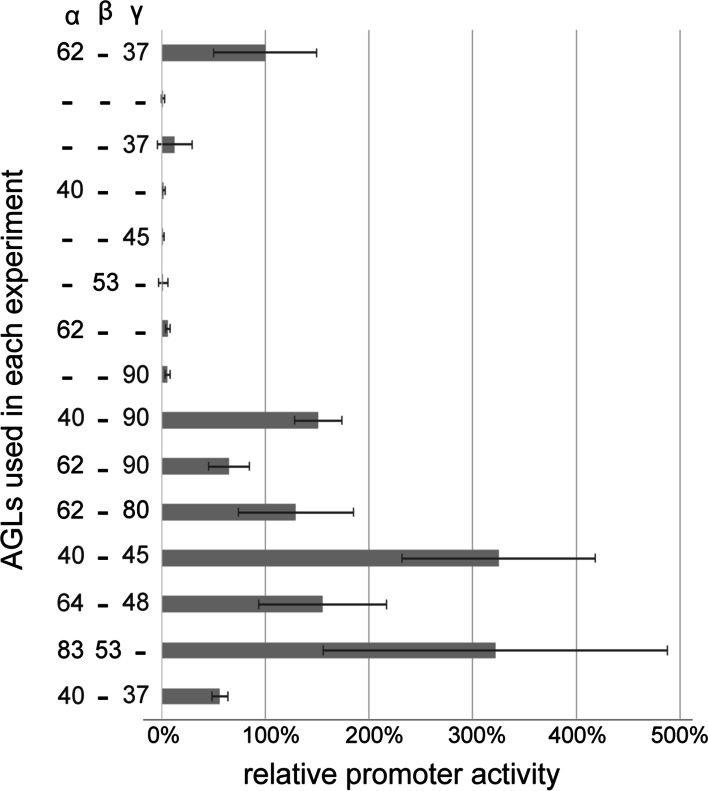
Relative InvINH1 promoter activity in protoplast transient expression assay. AGLs were tested for their ability to activate the InvINH1 promoter, either as monomers or as Mɑ-Mβ or Mɑ-Mγ dimers. C2 AGLs are in blue. Promoter activity was measured as the ratio of pInvINH1-GUS activity over luciferase activity (control for transfection efficiency). All promoter activities were normalized as the percentage of promoter activity in the presence of AGL62-AGL37 dimer. Averages and standard deviations were calculated from 2 to 16 biological replicates and two technical replicates for each biological sample

The type I MADS-box family is further classified into Mɑ, Mβ, Mγ, and Mδ subfamilies [34]. The majority of C2 AGLs have been shown to form a protein interaction network made of Mɑ-Mγ dimers [26]. This network is centered around AGL62 [26], which has a clear mutant phenotype with a severely shortened syncytial endosperm phase [21]. To test whether AGL62 regulates InvINH1 promoter activity by itself or in concert with other C2 AGLs, we first transfected Arabidopsis protoplasts with pInvINH1-GUS alone, or with pInvINH1-GUS and p35S-AGL62. Both experiments resulted in close to zero promoter activity, while protoplasts co-transfected with pInvINH1-GUS, p35S-AGL62 and p35S-AGL37 displayed significantly higher promoter activity (Fig. 1). Our data indicated that AGL62 and AGL37 form a dimer in the protoplast that directly activates the InvINH1 promoter.

Since InvINH1 expression is higher in the micropylar region and absent in the chalazal region of the syncytial endosperm [33], we next investigated whether this spatial specificity is due to the selective activation of InvINH1 by C2 AGLs that are preferentially expressed in the micropylar endosperm. For the purpose of comparison, all promoter activities were normalized as the percentage of promoter activation by the AGL62-AGL37 dimer. Our data indicated that C2 AGLs that are preferentially expressed in the micropylar endosperm, including AGL64, AGL48, and AGL90 [26], all activated the InvINH1 promoter in the form of a Mɑ-Mγ dimer (Fig. 1). However, InvINH1 promoter was also activated by the Mɑ-Mγ dimer containing C2 AGLs that are expressed in both the micropylar and chalazal endosperm (AGL40, AGL37), and by the Mɑ-Mγ dimer containing a C2 AGL that is expressed specifically in the chalazal endosperm (AGL45) (Fig. 1). Moreover, the InvINH1 promoter was also activated by the Mɑ-Mγ dimer formed by non-C2 AGLs (AGL62-AGL80) and by the Mɑ-Mβ dimer formed by non-C2 AGLs (AGL83-AGL53) (Fig. 1), suggesting that AGLs not regulated by the PRC2 complex are also capable of activating InvINH1 promoter in the protoplast.

Even though some AGL monomers also activated the InvINH1 promoter at low levels (1 to 13%, Fig. 1), the level of activation by AGL dimers was much higher, which ranged from 56% (AGL40-AGL37) to 325% (AGL40-AGL45) (Fig. 1). We also tested a transcription factor, ZHOUPI, which functions in the cellularized micropylar endosperm [6], and is unlikely to be an upstream regulator of InvINH1. The InvINH1 promoter activity in presence of ZHOUPI was 14 ± 2% (avg. ± std., data not shown). These data indicated that the activation of InvINH1 promoter in protoplast transient expression system relies on the presence of AGL dimer. However, we did not observe any correlation between InvINH1 promoter activity and the spatial expression patterns of AGLs. Collectively, our data suggested that there are additional regulators *in planta* that are responsible for the micropylar-preferred expression pattern of InvINH1.

### Two regions of the InvINH1 promoter are required for the AGL-mediated activation

**Fig. 2:**
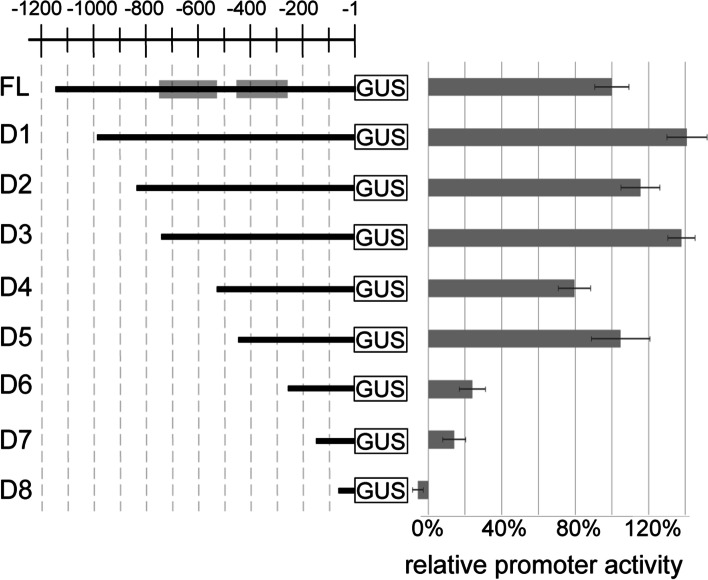
InvINH1 promoter deletion assay. A series of 5′ promoter deletion constructs (D1 to D8) was generated by making 100-200 bp deletions from the 5′ end of the full-length InvINH1 promoter (FL). Promoter activity in the presence of AGL40-AGL90 dimer was measured as the ratio of promoter-GUS activity over luciferase activity (control for transfection efficiency). All promoter activities were normalized as the percentage of full-length promoter activity in the presence of AGL40-AGL90 dimer. The shaded boxes on the full-length promoter indicate the location of promoter region 1 (− 741 to -524 bp) and region 2 (− 453 to -265 bp). Averages and standard deviations were calculated from 2 to 8 biological replicates and two technical replicates for each biological sample

To identify which region of the InvINH1 promoter is bound by the AGL dimer, we carried out promoter deletion analysis in the protoplast transient expression system. Out of the eight AGL dimers that activated the InvINH1 promoter in the protoplast assay system (Fig. 1), we selected the AGL40-AGL90 dimer for the promoter deletion analysis. Similar to InvINH1, both AGL40 and AGL90 are expressed in the micropylar endosperm and are controlled by the PRC2 complex [26]. Therefore, the AGL40-AGL90 dimer is more likely to activate InvINH1 *in planta* due to the overlap in their expression pattern. We generated eight InvINH1 promoter deletion constructs (D1 to D8) by deleting a 100-200 bp fragment at a time from the 5′ end of the InvINH1 full-length promoter (Fig. 2). Promoter activity was then analyzed in the presence of the AGL40-AGL90 dimer. Out of the eight promoter deletions we tested, two deletions (D4 and D6) led to significant reduction in promoter activity, where the reduction was more than 50% of the full-length promoter activity (Fig. 2). The D4 deletion (− 741 to -524 bp) reduced promoter activity from 138 ± 7% (D3) to 80 ± 9% (D4), while the D6 deletion (− 453 to -265 bp) reduced promoter activity from 105 ± 16% (D5) to 24 ± 7% (D6, Fig. 2). Our data suggested that InvINH1 promoter contains two regions (region 1: − 741 to -524 bp; region 2: − 453 to -265 bp) that are required for the AGL-mediated transcriptional activation in the protoplasts.

### The CArG sites within the enhancer 1 region are required for InvINH1 promoter activity

**Fig. 3:**
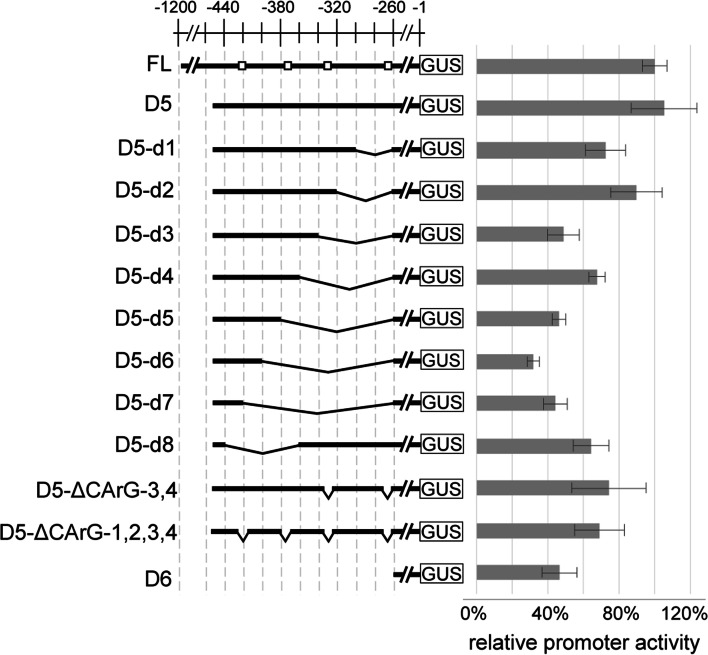
CArG sites are required for InvINH1 promoter activity. To delete the four putative CArG sites (denoted by open squares) located within the 189 bp enhancer 1 region (CArG1: -426 bp, CArG2: -374 bp, CArG3: -333 bp, and CArG4: -269 bp), a series of internal promoter deletion constructs were generated by making 20 bp sequential deletions from the 3′ end of enhancer 1 (D5-d1 to D5-d7), and by deleting an 80 bp region encompassing the CArG1 and CArG2 sites (D5-d8) from the D5 promoter fragment. D5-ΔCArG-3,4 and D5-ΔCArG-1,2,3,4 represent constructs in which only the CArG sites were specifically deleted. Promoter activity in the presence of AGL40-AGL90 dimer was measured as the ratio of promoter-GUS activity over luciferase activity (control for transfection efficiency). All promoter activities were normalized as the percentage of the full-length (FL) promoter activity in the presence of AGL40-AGL90 dimer. Averages and standard deviations were calculated from 4 to 32 biological replicates and two technical replicates for each biological sample

Prior research has shown that MADS-box transcription factors, including AGLs, have high binding affinity for the CArG consensus sequence, CC(A/T)_6_GG [28, 32, 35–37]. To determine whether the two promoter regions (− 741 to -524 bp; − 453 to -265 bp) contain CArG sites, we scanned the full-length InvINH1 promoter for the presence of CArG consensus sequence. Seven putative CArG sites (either C(A/T)_6_G or C(A/T)_7_G) were identified at locations -5 bp (CAAAAAATG), − 185 bp (CATTAAATG), − 269 bp (GCAAATATTGC), − 333 bp (CAATTTTG), − 374 bp (CTTAAAATG), − 426 bp (CTAAATTTG), and -966 bp (CAATAAAAG). Interestingly, four of these seven sites (CArG1: -426 bp, CArG2: -374 bp, CArG3: -333 bp, and CArG4: -269 bp) are located within the − 453 to -265 bp region, which was renamed as enhancer 1 (Fig. 3). To test whether the AGL40–90 dimer directly binds these four putative CArG sites, we made 20 bp sequential deletions from the 3′ end of enhancer 1 in the context of the D5 promoter fragment (D5-d1 to D5-d7), and an 80 bp internal deletion that removed the CArG1 and CArG2 sites (D5-d8, Fig. 3). The deletion of the CArG1 site led to an increase in promoter activity from 32 ± 3% to 44 ± 7% (D5-d6 vs. D5-d7), while the deletions of CArG2, CArG3, and CArG4 all led to a significant decrease in promoter activity, which changed from 68 ± 5% to 46 ± 4% for CArG2 (D5-d4 vs. D5-d5), from 90 ± 14% to 49 ± 9% for CArG3 (D5-d2 vs. D5-d3), and from 104 ± 16% to 72 ± 11% for CArG4 (D5 vs. D5-d1, Fig. 3). These data indicated that the three regions around the CArG2, CArG3, and CArG4 sites are required for InvINH1 promoter activity. To test whether the CArG sites themselves are required, we generated two constructs to delete just the CArG sequences from the D5 promoter fragment. Specific deletion of CArG3 and CArG4 reduced promoter activity from 104 ± 16% to 74 ± 21% (D5 vs. D5-ΔCArG-3,4), while the deletion of all four CArG sites reduced promoter activity from 104 ± 16% to 69 ± 14% (D5 vs. D5-ΔCArG-1,2,3,4, Fig. 3). Collectively, our data indicated that CArG2, CArG3, and CArG4 sites within the enhancer 1 region are required for the AGL-mediated activation of the InvINH1 promoter.

### Enhancer 1 is sufficient for AGL-mediated transcriptional activation

**Fig. 4:**
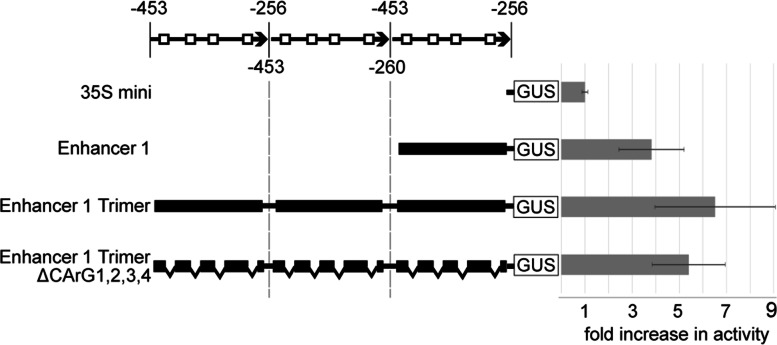
Enhancer 1 is sufficient to activate AGL-mediated transcription. A series of promoter constructs were generated by cloning the enhancer 1, a trimerized enhancer 1, and a trimerized enhancer 1 without the four CArG sites (ΔCArG 1,2,3,4) in front of the CaMV 35S minimal promoter. Promoter activity in the presence of AGL40-AGL90 dimer was measured as the ratio of promoter-GUS activity over luciferase activity (control for transfection efficiency). All promoter activities were normalized as fold increase over the 35S minimal promoter activity in the presence of AGL40-AGL90 dimer. The locations of CArG sites are denoted by open squares. Averages and standard deviations were calculated from 8 to 9 biological replicates and two technical replicates for each biological sample

To determine whether enhancer 1 alone is sufficient to recruit the AGL dimer to activate transcription, we cloned the 189 bp enhancer 1 sequence in front of the 35S minimal promoter. Promoter activity was then analyzed with protoplast transient assays in the presence of the AGL40-AGL90 dimer. The 35S minimal promoter alone displayed roughly 1% of the full-length InvINH1 promoter activity (data not shown). The addition of enhancer 1 led to 3.83 ± 1.39 fold increase in promoter activity over the 35S minimal promoter (Fig. 4). Since it has been shown that the binding affinity between MADS-box transcription factors and the promoter can be increased by concatemerizing regions of the promoter containing multiple CArG sites [37], we then tested whether trimerized enhancer 1 could lead to higher levels of transcriptional activation. The presence of the enhancer 1 trimer in front of the 35S minimal promoter led to 6.52 ± 2.56 fold increase in promoter activity, while the deletion of the four CArG sites from the enhancer 1 trimer reduced promoter activity from 6.52 ± 2.56 fold to 5.40 ± 1.55 fold (Fig. 4). However, this reduction in promoter activity is not significant due to the high background noise. Collectively, our data indicated that enhancer 1 containing CArG sites is sufficient to recruit the AGL40-AGL90 AGL dimer to activate transcription.

### Enhancer 1 is required to maintain InvINH1 expression in planta

To determine whether enhancer 1 is also required for InvINH1 expression *in planta*, we generated transgenic plants carrying the GFP reporter driven by the D5 or the D6 promoter fragment. Since transgene expression levels vary among independent transgenic lines, we used eight independent lines per construct to compare the GFP expression levels of the full-length [33], D5, and D6 promoter-GFP reporters (Additional file 1). GFP expression pattern was analyzed in 50 to 60 seeds per line at 2 days after pollination. Representative images were included in the Additional file 1. In D6 lines, the spatial distribution of GFP signal within the endosperm was similar to the pattern observed in full-length and D5 promoter lines (Additional file 1). However, the average GFP signal intensity was weaker in D6 lines when compared to the full-length and D5 lines (Additional file 1). These data indicated that enhancer 1 is required to maintain high levels of InvINH1 expression in the syncytial endosperm.

### AGL62 and FIS2 are required to regulate the expression of InvINH1 and InvINH2 in planta

**Fig. 5:**
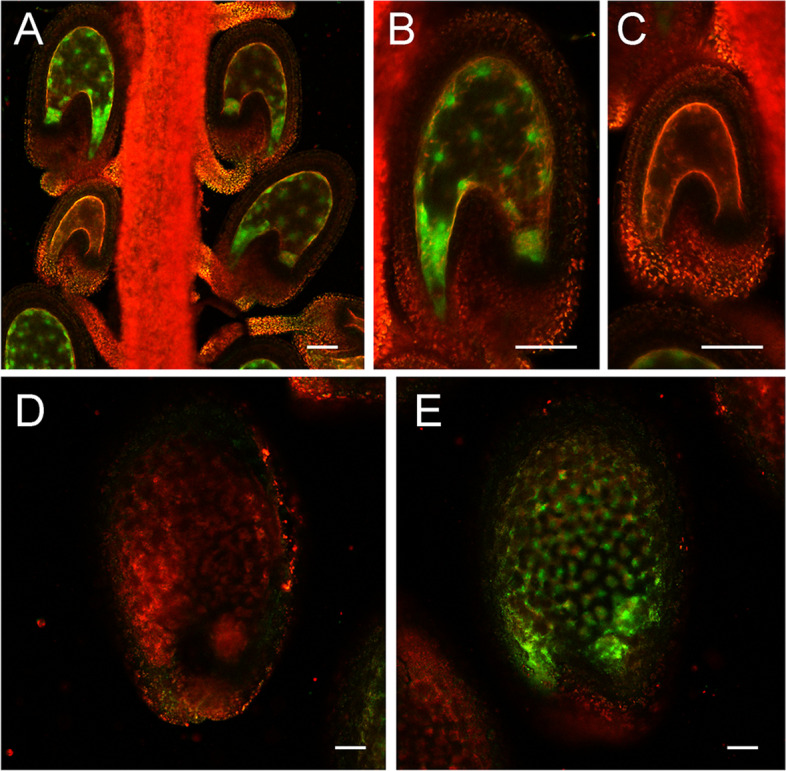
InvINH1 promoter-GFP activity in *agl62* and *fis2* mutants. Selfed seeds from a *agl62–1/+; T*^*GFP*^*/T*^*GFP*^ plant were imaged with a confocal microscope at pre-globular stage (A). The normal seeds (B) and the aborted seeds (C) from panel A were imaged again at higher magnification. The normal seeds (D) and the aborted seeds (E) from a selfed *fis2–8/+; T*^*GFP*^*/T*^*GFP*^ plant were imaged at early torpedo stage. The images in panel B to E were oriented with the micropylar ends on the left and the chalazal ends on the right. Bar = 50 μm

Our data from the protoplast transient expression assay indicated that AGL dimers bind the enhancer 1 region and activate the InvINH1 promoter. We next investigated whether AGLs are responsible for activating InvINH1 expression in the syncytial endosperm. In *agl62* heterozygous mutants, 25% of the self-pollinated seeds undergo precocious cellularization before endosperm stage VI, then abort later on [21]. Therefore, we tested whether InvINH1 promoter-GFP reporter is transcribed in *agl62* mutant seeds. A single-insertion line carrying the transgene for either InvINH1 or InvINH2 promoter-GFP reporter was crossed to either *agl62–1* or *agl62–2* mutant. Plants homozygous for the transgene and heterozygous for *agl62* mutant allele were identified from the second generation progenies. Both the InvINH1 and InvINH2 promoter-GFP reporters are expressed at the pre-globular stage [33]. However, around 25% of the seeds were GFP negative at pre-globular stage for both *agl62–1/+;T*^*GFP*^*/T*^*GFP*^ and *agl62–2/+;T*^*GFP*^*/T*^*GFP*^ plants (chi-square *P* > 0.05 for 3:1 segregation test, Table 1, Fig. 5A-C). These data indicated that InvINH1 and InvINH2 were not expressed in the *agl62* homozygous mutant seeds.

**Table 1:** Both FIS2 and AGL62 regulate the expression of InvINH1 and InvINH2 *in planta*

genotype	GFP(+)	GFP(−)	total number of seeds	expected ratio	Chi-square test ***P*** value
pInvINH1-GFP/pInvINH1-GFP; agl62–1/+	123	43	166	3:1	0.788
pInvINH1-GFP/pInvINH1-GFP; agl62–2/+	167	58	225	3:1	0.788
pInvINH2-GFP/pInvINH2-GFP; agl62–1/+	131	42	173	3:1	0.826
pInvINH2-GFP/pInvINH2-GFP; agl62–2/+	165	58	223	3:1	0.728
pInvINH1-GFP/pInvINH1-GFP; fis2–8/+	78	75	153	1:1	0.808
pInvINH2-GFP/pInvINH2-GFP, fis2–8/+	69	73	142	1:1	0.737

Segregation analysis was used to confirm that all the plants in this study are homozygous for the promoter-GFP transgene. Promoter-GFP expression was analyzed at pre-globular stage and at early torpedo stage for *agl62/+* mutant and *fis2/+* mutant, respectively

The FIS-PRC2 complex has been shown to suppress the expression of type I MADS box genes [26]. In *fis2/+* plants, 50% of self-pollinated seeds fail to cellularize and abort at heart stage [26]. Therefore, we next investigated whether the InvINH1 and InvINH2 promoter-GFP reporters are up-regulated in *fis2* mutant seeds. A single-insertion line carrying the transgene for either the InvINH1 or InvINH2 promoter-GFP reporter was crossed to *fis2–8* mutant. Plants homozygous for the transgene and heterozygous for *fis2* mutant allele were identified from the second generation progenies. The InvINH1 and InvINH2 promoter-GFP reporters are not expressed at the early torpedo stage [33]. However, in *fis2–8/+;T*^*GFP*^*/T*^*GFP*^ plants, around 50% of the seeds were GFP positive at early torpedo stage (chi-square *P* > 0.05 for 1:1 segregation test, Table 1, Fig. 5D-E), indicating that FIS2 is required to suppress the expression of InvINH1 and InvINH2 in the cellularized endosperm, likely via the suppression of AGLs.

## Discussions

Our prior work indicated that a putative invertase inhibitor, InvINH1, suppresses embryo growth rate before endosperm cellularization [33]. InvINH1 is preferentially expressed in the micropylar endosperm that surrounds the embryo, and is quickly down-regulated after endosperm cellularization [33]. In this study, we identified a group of type I MADS box transcription factors (AGLs) as the upstream regulators that activate InvINH1 expression in the syncytial endosperm. We also identified an enhancer region in the InvINH1 promoter that contains the cis-elements for AGLs. Moreover, InvINH1 is not expressed in *agl62* mutant seeds, indicating that AGL62 is a key regulator for activating InvINH1. Since AGL62 is a downstream target repressed by the FIS-PRC2 complex [20], our findings provided the missing link between InvINH1 and FIS2, which could explain the apparent up-regulation of InvINH1 in *fis2* mutant (Fig. 5) [33].

Most of the AGLs identified in this study have been shown to be down-regulated after endosperm cellularization [26], which could explain the preferential expression of InvINH1 in the syncytial endosperm. However, we have yet to identify the upstream regulators of InvINH1 that are responsible for the micropyle-preferred expression of InvINH1. In our protoplast assay system, both micropyle-preferred and chalaza-preferred AGLs are capable of activating the InvINH1 promoter (Fig. 1), even though InvINH1 is not expressed in the chalazal endosperm [33]. This discrepancy could be explained if there are transcriptional repressors of InvINH1 that are specifically expressed in the chalazal endosperm and are absent in leaf mesophyll protoplasts. This scenario is possible because isolated protoplasts tend to retain their original cell fate, which means endosperm-specific genes are not expressed in isolated protoplasts [38].

Data from our promoter deletion assay indicated that InvINH1 is regulated by both transcriptional repressors and activators. Besides the two promoter deletions that resulted in a decrease in InvINH1 promoter activity (D4: − 741 to -524 bp, and D6: − 453 to -265 bp), there are three promoter deletions (D1: − 1141 to -997 bp, D3: − 834 to -742 bp, and D5: − 523 to -454 bp) that resulted in an increase in promoter activity (Fig. 2), suggesting the presence of silencer sequences in these three regions of the InvINH1 promoter. Therefore, there is a possibility that the transcriptional repressors interacting with these silencers may be involved in the suppression of InvINH1 in the chalazal endosperm.

Interestingly, some MADS box transcription factors could also act as transcriptional repressors [39–43]. We detected seven putative CArG sites in the InvINH1 promoter, four of which are located in the enhancer 1 region. Three of these four CArG sites were confirmed by our protoplast assay as the binding sites for AGLs (Fig. 3). The MADS box proteins often form higher-order heterotetrameric complexes that are recruited to the target promoter via multiple CArG sites [30, 31]. Even though CArG sites are prevalent in the Arabidopsis genome and nearly present in every gene [29], the number of CArG sites and the sequence context in the promoter could determine the composition of the heterotetrameric complexes, which in turn could recruit different types of regulators to the target promoter, such as transcriptional activators, repressors, and chromatin remodeling complexes [30, 31]. It is not clear whether type I MADS box proteins could form heterotetrameric complexes similar to type II MADS box proteins. However, the potential difference in composition between the MADS-box complexes formed in the endosperm and the protoplast could also explain why the chalaza-preferred AGLs could activate the InvINH1 promoter in the protoplast but not in the chalazal endosperm.

Micropylar endosperm and chalazal endosperm have distinct functions during seed development. However, the molecular mechanism underlying endosperm differentiation has not been fully elucidated. Our prior work has shown that InvINH1 is preferentially expressed in the micropylar endosperm to regulate embryo growth rate [33]. In this study, we discovered several type I MADS box genes as the upstream activators of InvINH1. Our data also suggested that these AGLs serve as the intermediate step during the FIS2-PRC2-mediated suppression of InvINH1 upon endosperm cellularization (Fig. 5). Our findings agree with a chromatin immunoprecipitation study that has identified more than a thousand direct targets of AGL37/PHE1, including both InvINH1 and InvINH2 [32]. Unlike type II MADS box genes, there are fewer studies on type I MADS box genes probing the structural basis of their target specificity. Future work in this area has the potential to reveal the function of endosperm-specific type I MADS box genes during endosperm differentiation and their connection to endosperm cellularization, which is an important developmental transition mediated by the FIS2-PRC2 complex.

## Conclusions

Our prior publication indicated that the micropylar endosperm produces a putative invertase inhibitor (InvINH1) to suppress embryo growth during the syncytial phase. In this study, we discovered that a group of type I MADS-box transcription factors form dimers to activate InvINH1 promoter. Even though type I MADS-box genes are already known to form dimers in yeast two-hybrid system, our study is the first one to demonstrate that the dimers formed by type I MADS-box genes are capable of activating transcription in plant cells. Some of the type I MADS-box genes identified in this study are the known targets of the FIS-PRC2 complex, which is a chromatin remodeling complex required for endosperm cellularization. Collectively, our findings revealed a regulatory pathway that is responsible for activating InvINH1 in the syncytial endosperm, and for down-regulating InvINH1 after endosperm cellularization. This specific expression pattern of InvINH1 is likely responsible for the observed difference in embryo growth rate before and after endosperm cellularization in Arabidopsis.

## Methods

### Plant materials and growth conditions

Seeds for wild type *Arabidopsis thaliana* plants (ecotype Col-0) and *fis2–8* mutant [44] were obtained from Ramin Yadegari’s lab at the University of Arizona. Seeds for *agl62–1* (SALK_137707) and *agl62–2* (SALK_022148) mutants [21] were obtained from Arabidopsis Biological Resource Center. Permissions were not necessary to collect the seed samples described above. All plants were grown as previously described [33]. In brief, seeds were stratified at 4 °C in the dark for 3–4 days, then planted in 2-in. pots filled with Pro-Mix BX soil (Premier Horticulture). Seedlings were kept under humidity domes for 1 week after germination. All plants used in this study were grown in a walk-in Environmental Room (Norlake Scientific) at 22 °C. Plants were watered three times per week and fertilized once a week with an all-purpose 20–20-20 fertilizer (Scotts-Sierra Horticultural Products Company). Plants used for protoplast isolation were grown under short-day condition (12-h light/12-h dark) with 50-75 μmol·m^− 2^·s^− 1^ light intensity. The rest of the plants were grown under long-day condition (16-h light/8-h dark) with 180–200 μmol·m^− 2^·s^− 1^ light intensity.

### Plasmid construction

The constructs for overexpressing AGL (pUC19-d35Stev-AGL) in the protoplast transient expression assay were created by cloning the respective AGL coding region downstream of a double 35S promoter and a translational enhancer from tobacco etch virus in a pUC19 backbone [45]. Since the AGL genes used in this study don’t contain any introns, the coding region of AGLs were directly amplified from Col-0 genomic DNA with the iProof high-fidelity DNA polymerase (Fisher Scientific), then cloned into pUC19-d35Stev-GFP (GenBank accession MT647188) between two restriction sites (BamHI and KpnI for AGL36, AGL37, AGL40, AGL45, AGL53, AGL62, AGL80, AGL83, and AGL90; XbaI and KpnI for AGL48 and AGL64), replacing the GFP coding region. Due to high sequence homology to other AGLs, a nested PCR was used to amplify AGL53. The coding region of ZHOUPI (negative control) was amplified from the cDNA prepared from Col-0 siliques harvested at 5 days after pollination, then cloned into pUC19-d35Stev-GFP between BamHI and KpnI site. The preparation of cDNA has been described previously [33]. The primers used for all PCR reactions are listed in Additional file 2.

To construct the full-length InvINH1 promoter-GUS reporter (pUC-pInvINH1-GUS) used in the protoplast transient expression assay, the 1172 bp 5′ flanking region of InvINH1, including the entire 5′ intergenic region and the coding region for the first seven amino acids, was subcloned from pBN-pInvINH1-GFP [33] into pBI101 (Clontech) between the XbaI and the BamHI sites, resulting in pBI-pInvINH1-GUS. The HindIII/EcoRI fragment containing the InvINH1 promoter, GUS coding region, and the Nos terminator sequence was then subcloned from pBI-pInvINH1-GUS into pUC19 [45], resulting in pUC-pInvINH1-GUS. To generate the eight constructs for deleting the 5′ end of the full-length InvINH1 promoter in 100-200 bp increments (pUC-pInvINH1^D1^-GUS to pUC-pInvINH1^D8^-GUS), fragments of the InvINH1 promoter were amplified from pUC-pInvINH1-GUS with the iProof high-fidelity DNA polymerase (Fisher Scientific) using the primers listed in Additional file 2, then cloned into pUC-pInvINH1-GUS between XbaI and BamHI site, replacing the full-length InvINH1 promoter.

A Q5 mutagenesis kit (New England Biolabs) was used to generate internal deletions in the InvINH1 promoter D5 fragment in 20 bp increments (pUC-pInvINH1^D5d1^-GUS to pUC-pInvINH1^D5d8^-GUS), or to delete the CArG sites from the InvINH1 promoter D5 fragment (pUC-pInvINH1^D5𝝙CArG3,4^-GUS and pUC-pInvINH1^D5𝝙CArG1,2,3,4^-GUS). The internal deletions were generated by amplifying pUC-pInvINH1^D5^-GUS through inverse PCR with non-overlapping primers followed by ligation. All mutagenesis reactions (PCR, ligation, and *E.coli* transformation) were carried out following the manufacturer’s instructions. All the primers used in mutagenesis PCR are listed in Additional file 2.

To clone the enhancer 1 upstream of the 35S minimal promoter, we first generated the construct pUC-35S(− 90)-TEV-GUS. Briefly, a region including the CaMV 35S minimal promoter (− 90 bp) and the TEV translational enhancer was PCR amplified from pUC19-d35Stev-GFP, then cloned into pUC-pInvINH1-GUS between XbaI and BamHI site, replacing the InvINH1 promoter. The enhancer 1 monomer was then PCR amplified from pUC-pInvINH1^D5^-GUS and cloned into pUC-35S(− 90)-TEV-GUS between the HindIII and XbaI sites. To construct the enhancer 1 trimer, the enhancer 1 region (wildtype or 𝝙CArG) was first PCR-amplified from the pUC-pInvINH1^D5^-GUS or pUC-pInvINH1^D5𝝙CArG1,2,3,4^-GUS as separate HindIII-XbaI, XbaI-BamHI, and BamHI-PstI fragments, then digested and ligated to form the enhancer 1 trimer, which was cloned into pUC-35S(− 90)-TEV-GUS between HindIII and PstI site. The resulting trimers had 6 bp linkers between each enhancer 1 repeat. To include the entire CArG4 sequence, the enhancer 1 region (− 453 to -256 bp) used in these constructs was expanded from -265 bp to -256 bp. The primers used for all PCR reactions are listed in Additional file 2.

Constructs used for plant transformation were generated by cloning fragments of the InvINH1 promoter into vector pBN-GFP [44]. The InvINH1 promoter D5 fragment and D6 fragment were amplified from pBN-pInvINH1-GFP [33] with the iProof high-fidelity DNA polymerase (Fisher Scientific) using primers listed in Additional file 2. The amplified InvINH1 promoter fragment was then cloned into pBN-GFP [44] between BamHI and XbaI site. All the constructs generated in this study were verified by Sanger sequencing (Eurofins).

### Protoplast isolation and transformation

Arabidopsis mesophyll protoplasts were isolated from the rosette leaves of four to five-week-old Col-0 plants using modified versions of two published protocols [46, 47]. In brief, the lower epidermal layer of each leaf was removed by placing the leaf on a flat work surface with the abaxial side facing up. The leaves were secured to the surface using a piece of single-sided Scotch clear tape (3 M Corporate). By carefully pulling the tape off, the lower epidermal layer was removed from the leaves, exposing the mesophyll cells. The stripped leaves were submerged in a petri dish filled with filter-sterilized enzyme solution containing 1.5% cellulase “onozuka” R-10, 0.4% macerozyme R-10, 20 mM MES (pH 5.7), 20 mM KCl, 0.4 M mannitol, 10 mM CaCl_2_, 0.1% BSA, and 1 mM β-Mercaptoethanol (14.3 M), then vacuum-infiltrated for 30 min to remove residual air bubbles. The petri dish was then incubated for 2.5 h at 21 °C in the dark. Afterwards, the protoplasts were released by gentle shaking. The protoplast suspension was filtered with a 40-μm nylon cell strainer (Corning). After two washes with ice-cold W5 solution (154 mM NaCl, 125 mM CaCl_2_, 5 mM KCl, 2 mM MES, pH 5.7), protoplast density was determined using a hemocytometer, then adjusted to 2 × 10^5^ protoplasts / mL in MMg solution (0.4 M mannitol, 15 mM MgCl_2_, 4 mM MES, pH 5.7).

Each protoplast transfection reaction contained 100 μL protoplast suspension (2 × 10^5^ protoplasts / mL) and 10uL mixed plasmid DNA solution (14.4 μg). The plasmid DNA mixture was made of 0.4 μg pUC19-35S-LUC [48], 4 μg promoter-GUS reporter construct, 5 μg pUC19-d35Stev-AGL or pUC19-d35Stev-ZHOUPI, and sufficient amounts of pUC19 so that the total amount of plasmid DNA is 14.4 μg in each reaction. The protoplasts were gently mixed with the plasmid solution. Transfections were facilitated by adding 110 μL of a 40% PEG solution (40% PEG4000, 0.2 M mannitol, 0.1 M CaCl_2_), mixing by gently inverting the tubes four times and incubating at room temperature for 10 min. Reactions were stopped by adding 880 μL W5. The protoplasts were spun down and resuspended in 200 μL W5. Protoplasts were added to 6-well plates coated with 5% calf-serum containing 800 μL W5 solution and incubated at 21 °C in the dark for 19 h. After incubation, protoplasts were pelleted at low speed and frozen in liquid nitrogen. Samples were stored at − 80 °C until further analysis. Two biological replicates were run sequentially at a time. The total number of biological replicates used to calculate each data point was described in figure legends.

### Luciferase and GUS fluorescence assays

Luciferase and GUS assay protocols were adapted from published protocols [49, 50]. Briefly, protoplast pellets were thawed on ice and lysed with 50 μL Glo Lysis Buffer (Promega). Samples were vortexed for 10s and incubated on ice for 10 m. The Bright-Glo Luciferase (Promega) reagent was stored at − 80 °C after reconstitution and diluted 1:4 before use. After mixing 10 μL cell lysate with 100 μL diluted luciferase reagent, the mixture was analyzed on a Synergy™ H1MD Microplate reader (BioTek) with the following settings: Emission, Full light; Optics, Top; Gain, 100. Reactions with luciferase reading below 200 were considered as failed reactions, which were removed from the dataset. Each lysate reaction was duplicated to produce two technical replicates for each biological replicate. Two technical replicates were also carried out for the no-cell-lysate control reaction to account for background noise.

For the GUS fluorescence assay, 2 μL protoplast lysate was mixed with 25 μL MUG substrate solution (1 mM MUG, 10 mM Tris, pH 8.0, 2.5 mM MgCl_2_) and incubated in the dark for 1 h at 37 °C. The reaction was stopped with 100 μL 0.2 M Na_2_CO_3_ and analyzed on a Synergy™ H1MD plate reader (BioTek) with the following settings: Excitation, 360 nm; Emission, 460 nm; Optics, Top; Gain, 50; Light Source, Xenon Flash; Lamp Energy, High. Each cell lysate was used in two duplicate reactions to act as technical replicates. Two technical replicates were also carried out for the no-cell-lysate control reaction to account for background noise.

### Statistical analysis of protoplast transient expression data

Promoter activity was calculated using the formula (GUS_experiment_ - GUS_control_) / LUC_experiment_, since the value of LUC_control_ was close to zero. For graphing purposes, relative promoter activity was reported as a percentage of the reference promoter activity. The data point used as the reference point was described in figure legends. The average and standard deviation for each data point were calculated using Excel.

### Plant transformation and transgenic plant selection

Transgenic Arabidopsis plants were produced using the standard floral dip method [51]. To summarize, binary constructs of interest containing a kan selection marker were introduced into *Agrobacterium tumefaciens* strain GV3101 pMP90 [52]. A 50 mL bacterial culture was grown at 28 °C to an O.D. (optical density) between 1.5–2.0. Cells were pelleted at room temperature and resuspended in infiltration medium (0.5x Murashige and Skoog (MS) salts, 5% sucrose, 1x Gamborg’s B5 vitamin stock (Bioworld), 0.25 mM Silwet, pH adjusted to 5.0 with KOH) to an O.D. between 1.5–2.0. The aerial parts of flowering Arabidopsis plants were dipped in the infiltration medium and left at 21 °C in the dark overnight to recover. Dipped plants were kept under long-day conditions (16-h light/8-h dark) to set seeds. Around 10 plants were dipped in total for each transformation.

T1 seeds were sterilized using 70% EtOH and bleach, then suspended in 0.1% low melting-point agarose and plated on 0.5x MS media containing 35 μg/mL kanamycin and 50 μg/mL cefotaxime. Plates were kept at 4 °C in the dark for 3 days, then incubated in a growth chamber (Percival Scientific CU36L5) at 22 °C under long-day conditions (16-h light/8-h dark with 80–100 μmol·m^− 2^·s^− 1^ light intensity) for 7–10 days, or until the green seedlings were large enough to be transferred to soil. After transplantation, seedlings were kept in the dark overnight before being transferred to standard growing conditions.

### Genotyping transgenic and mutant plants

Genomic DNA was extracted from leaf tissue using glass bead maceration, according to published protocols [53]. To summarize, a large rosette leaf was placed in a 1.5 mL microcentrifuge tube along with 100 μL 1 mm zirconia glass beads (RPI). The sample was flash-frozen in liquid nitrogen and pulverized using a Silamat Plus tissue lyser (Vivadent) by pulsing for 9 s. Ground samples were suspended with 400 μL extraction buffer (200 mM Tris-HCl (pH 7.5), 250 mM NaCl, 25 mM EDTA, 0.5% SDS) and pulsed again for 9 s. Genomic DNA was precipitated from the supernatant with 600 μL EtOH at room temperature for 2 min. The DNA pellets were washed with 500 μL 70% EtOH, air dried, then resuspended in 10 μL of 10 mM Tris buffer (pH 8.0) at 4 °C overnight. Genotyping PCR was carried out using the appropriate primers listed in Additional file 2.

### Image collection and processing

Developing seeds were dissected from the siliques at globular or early torpedo stage as described previously [54, 55]. Briefly, siliques were held in place with forceps and opened lengthwise with dissecting needles under a Motic SMZ-171 dissecting microscope (Motic). Dissected seeds were imaged either with a LSM 700 inverted confocal microscope (Zeiss), or with a Nikon Eclipse E1000 epifluorescence microscope equipped with a GFP bandpass filter (exciter 457-487 nm; emitter 502-538 nm, Nikon) and a Moticam 1080 digital camera (Motic). Images were processed using Adobe Photoshop CS (Adobe Systems Inc).

## Supplementary Information

**Additional file 1.**

**Additional file 2.**

## Data Availability

All generated or analyzed data were included in this published article.

## Abbreviations

Col-0: Columbia-0
InvINH1: Invertase inhibitor 1
InvINH2: Invertase inhibitor 2
AGL: AGAMOUS-LIKE
PRC2: Polycomb Repressive Complex 2
