# Supplementary material for: The identification of type I MADS box genes as the upstream activators of an endosperm-specific invertase inhibitor in Arabidopsis
Source: BMC Plant Biol. 2022 Jan 6;22:18. doi: 10.1186/s12870-021-03399-3 (PMC8734259; doi:10.1186/s12870-021-03399-3)
Supplement: Supplementary file 1 — Additional file 1. [file 12870_2021_3399_MOESM1_ESM.pdf]

Supplemental Data

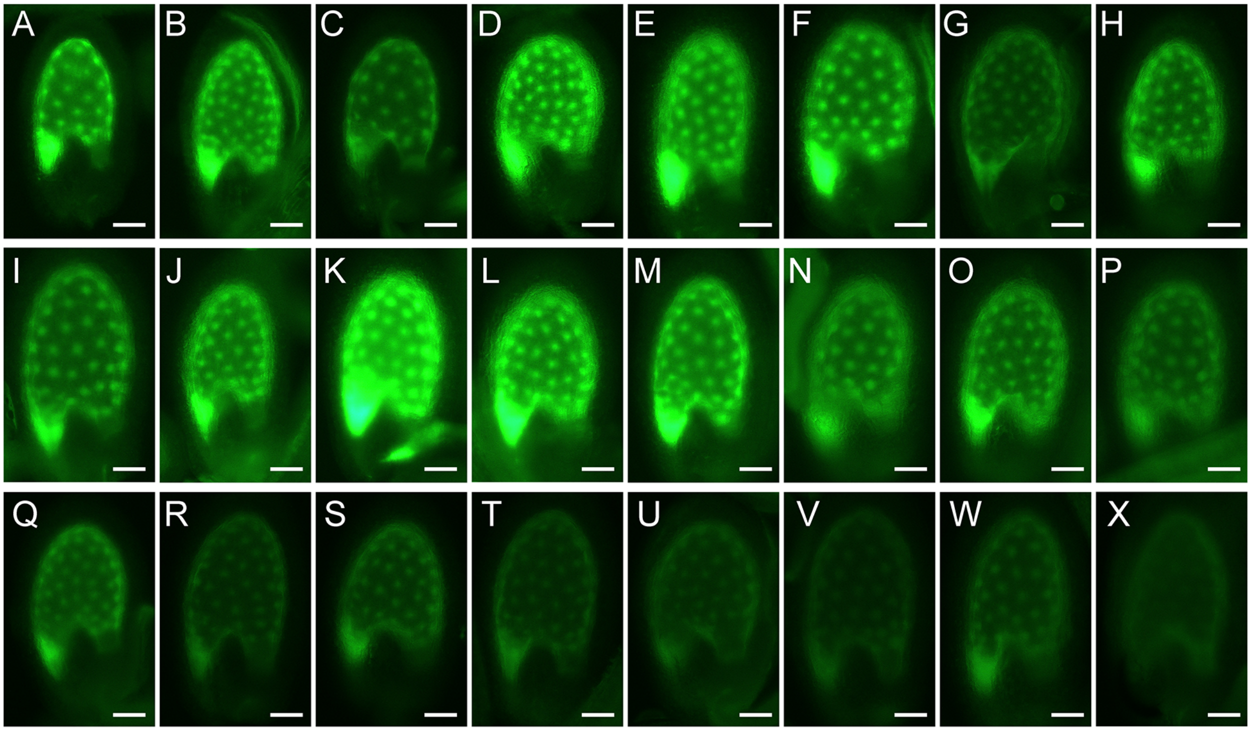

Figure S1. The comparison of promoter-GFP activity in the endosperm between transgenic lines carrying transgene pInvINH1-GFP (A-H), pInvINH1-D5-GFP (I-P) and pInvINH1-D6-GFP (Q-X). Selfed seeds from either T2 or T3 plants representing eight independent T1 lines for each construct were imaged with an epifluorescence microscope around endosperm stage VII (50 nuclei) (Boisnard-Lorig et al., 2001). All the images are oriented with the micropylar ends on the left and the chalazal ends on the right. Bar = 50 µm.

References

Boisnard-Lorig, C., Colon-Carmona, A., Bauch, M., Hodge, S., Doerner, P., Bancharel, E., Dumas, C., Haseloff, J., and Berger, F. (2001). Dynamic analyses of the expression of the HISTONE::YFP fusion protein in arabidopsis show that syncytial endosperm is divided in mitotic domains. Plant Cell *13*, 495–509.
