# Supplementary material for: The identification of type I MADS box genes as the upstream activators of an endosperm-specific invertase inhibitor in Arabidopsis
Source: BMC Plant Biol. 2022 Jan 6;22:18. doi: 10.1186/s12870-021-03399-3 (PMC8734259; doi:10.1186/s12870-021-03399-3)
Supplement: Supplementary file 2 — Additional file 2. [file 12870_2021_3399_MOESM2_ESM.pdf]

**Table S1. Primer List**

| Description | Gene/Fragment | Forward Primer | | | Reverse Primer | | |
|---|---|---|---|---|---|---|---|
| | | Primer Name | Sequence | Restriction Site | Primer Name | Sequence | Restriction Site |
| | AGL36 | AGL36-cds-F | CAGGATCCATGAAGAAGGTGAAGCTATCTTTG | BamHI | AGL36-cds-R | CTGGTACCTTAGAGATTATTGTTGATGTAAGG | KpnI |
| | AGL37 | AGL37-cds-F | TAGGATCCATGAGGGGGAAGATGAAGTTATCG | BamHI | AGL37-cds-R | GTGGTACCCTAGAGATCATTGATGATGTTAGG | KpnI |
| | AGL40 | AGL40-cds-F | TAGGATCCATGGTGAGAAGTACCAAAG | BamHI | AGL40-cds-R | TCGGTACCCTAGCTCTGGTTGAAGTTG | KpnI |
| | AGL45 | AGL45-cds-F | TAGGATCCATGACGAGGAAGAAGCTAAA | BamHI | AGL45-cds-R | TCGGTACCCTAGGGTTGTTGGTAGTAAAAG | KpnI |
| | AGL53 nested PCR | AGL53-nested-F | GGATGTGGGAAATCGTATCTAAGATCG | | AGL53-nested-R | AGAATCGGGTAAAACGAGATAAGCAAC | |
| | AGL53 | AGL53-cds-F | ATGAGGATCCATGGATTCTTCAATGTCGACGAAGA | BamHI | AGL53-cds-R | ATGAGGTACCCTAATTATAAGGGAACATCATTGGATCTGC | KpnI |
| | AGL62 | AGL62-cds-F | CTGGATCCATGGTGAAAAAAAGCAAAGGTCGTC | BamHI | AGL62-cds-R | CAGGTACCTTAATAGTAATCAGATCTAGACTGG | KpnI |
| | AGL80 | AGL80-cds-F | ATGAGGATCCATGACAAGAAAGAAAGTGAAACTTGC | BamHI | AGL80-cds-R | ATGAGGTACCCTAATGGAACCATGTTTTATTGGTAATACT | KpnI |
| | AGL83 | AGL83-cds-F | ATGAGGATCCATGAGATTCGTTCCTTATTTATACGAG | BamHI | AGL83-cds-R | ATGAGGTACCTCATATACTTCTTCTTCGTTTTGCATAAGA | KpnI |
| | AGL90 | AGL90-cds-F | GTGGATCCATGAAGAAGGTAAAGCTATCTTTG | BamHI | AGL90-cds-R | CAGGTACCTTAGAGATTGTTGTTGATGTAAGG | KpnI |
| | AGL48 | AGL48-cds-F | TATCTAGAATGACAAGAAAGAAAGTAAAAC | XbaI | AGL48-cds-R | GTGGTACCTTAGAAATGATGATTGTTAAAAC | KpnI |
| Amplify AGL coding region | AGL64 | AGL64-cds-F | TATCTAGAATGAAACCCAAGAAAACCAA | XbaI | AGL64-cds-R | CTGGTACCTCATTTGTTATTATTCTCATCATGTC | KpnI |
| Amplify ZHOUPI coding region | ZHOUPI | ZHOUPI-cds-F | TATTAGGATCCATGACTAATGCTCAAGAGTTG | BamHI | ZHOUPI-cds-R | ATTAGGTACCTTATAGAGATGAAAAATATAACACCAG | KpnI |
| amplify InvINH1 full-length promoter (Zuma et al. 2018) | pInvINH1 | InvINH1_F | AATGTCTAGAGCTGAAATGAAACTACATGTGC | XbaI | InvINH1_R | GAGAAGGATCCCAATGAAACCAAGAACTTCAT | BamHI |
| | pInvINH1-D1 | InvINH1-D1_F | TTACTCTAGATTGAAACACAGGGTGACCA | XbaI | InvINH1_R | same as above | |
| | pInvINH1-D2 | InvINH1-D2_F | TGGATCTAGATCTATTTCAAATTGGGAACTTTTCCA | XbaI | InvINH1_R | same as above | |
| | pInvINH1-D3 | InvINH1-D3_F | AAAGTCTAGATTGCATGTTGACCAATTCCA | XbaI | InvINH1_R | same as above | |
| | pInvINH1-D4 | InvINH1-D4_F | TTAATCTAGAGATTTTGGAAACACTCACAATCGT | XbaI | InvINH1_R | same as above | |
| | pInvINH1-D5 | InvINH1-D5_F | CAAATCTAGATGAATGCATAATGATGATGAC | XbaI | InvINH1_R | same as above | |
| | pInvINH1-D6 | InvINH1-D6_F | GAACTCTAGATGCACAATGGAATGGAGAGTTGCTA | XbaI | InvINH1_R | same as above | |
| | pInvINH1-D7 | InvINH1-D7_F | TACATCTAGACCAGACCAGAAACAACAGCAAGAA | XbaI | InvINH1_R | same as above | |
| 5' deletion of InvINH1 full-length promoter in 100bp increments | pInvINH1-D8 | InvINH1-D8_F | CCGTTCTAGACCTTCCGTATTACAAAGACCGAA | XbaI | InvINH1_R | same as above | |
| | pInvINH1-D5-d1 | InvINH1-D5-d1_F | TTGCATGTTGACCAATTC | n/a | InvINH1-D5-d1_R | TTCTAAAAATCATTCATACTTTCAAAATTG | n/a |
| | pInvINH1-D5-d2 | InvINH1-D5-d1_F | same as above | n/a | InvINH1-D5-d2_R | TTCAAAATTGATATCTCTTCTAC | n/a |
| | pInvINH1-D5-d3 | InvINH1-D5-d1_F | same as above | n/a | InvINH1-D5-d3_R | TACCATTATGCTATTTGAAC | n/a |
| | pInvINH1-D5-d4 | InvINH1-D5-d1_F | same as above | n/a | InvINH1-D5-d4_R | AACATTTTAAGAAAAATCAAAGATTATTTG | n/a |
| | pInvINH1-D5-d5 | InvINH1-D5-d1_F | same as above | n/a | InvINH1-D5-d5_R | AGATTATTTGATTTATTTGTTTTCC | n/a |
| | pInvINH1-D5-d6 | InvINH1-D5-d1_F | same as above | n/a | InvINH1-D5-d6_R | TTTCCATATATTCACAAATTTAGTC | n/a |
| | pInvINH1-D5-d7 | InvINH1-D5-d1_F | same as above | n/a | InvINH1-D5-d7_R | TAGTCAACGATTGTGAGTG | n/a |
| Internal deletion of InvINH1 D5 fragment in 20bp increments | pInvINH1-D5-d8 | InvINH1-D5-d8_F | GAAGAGATATCAATTTTGAAAGTATG | n/a | InvINH1-D5-d8_R | TTCCAAAATCTCTAGAGTC | n/a |
| | pInvINH1ΔCArG-1 | CArG-1-del-F | TGAATATATGGAAAACAAATAAATCAAATAATCT | n/a | CArG-1-del-R | TCAACGATTGTGAGTGTTTC | n/a |
| | pInvINH1ΔCArG-2 | CArG-2-del-F | TTGTTCAAATAGCATAATGGTAGAAG | n/a | CArG-2-del-R | AAAAATCAAAGATTATTTGATTTATTTGTTTTC | n/a |
| | pInvINH1ΔCArG-3 | CArG-3-del-F | AAAGTATGAATGATTTTTAGAAAATGGAA | n/a | CArG-3-del-R | ATATCTCTTCTACCATTATGCTATTTG | n/a |
| generate CArG-site deletions | pInvINH1ΔCArG-4 | CArG-4-del-F | CATGTTGACCAATTCCAAAC | n/a | CArG-4-del-R | CTTTCCATATACGCACGT | n/a |
| amplify 35S minimal promoter | 35S(-90)-TEV | 35Smini(-90)-TEV-F | ATTATCTAGAATCTCCACTGACGTAAGGG | XbaI | 35Smini(-90)-TEV-R | ATTAGGATCCATCGTTCGTAAATGGTGAAAATTTTC | BamHI |
| | enhancer 2 monomer | InvINH1-D5_F_HindIII | AATAAAGCTTTGATTTTGGAAACACTCACAATCGT | HindIII | InvINH1-D6_R_XbaI | ATTATCTAGACAGGCAATATTTGCTTTCCATATACG | XbaI |
| | enhancer 2-HindIII/XbaI | InvINH1-D5_F_HindIII | AATAAAGCTTTGATTTTGGAAACACTCACAATCGT | HindIII | InvINH1-D6_R_XbaI | ATTATCTAGACAGGCAATATTTGCTTTCCATATACG | XbaI |
| | enhancer 2-XbaI/BamHI | InvINH1-D5_F | TTAATCTAGAGATTTTGGAAACACTCACAATCGT | XbaI | InvINH1-D6_R_BamHI | TTAAGGATCCCAATATTTGCTTTCCATATACGCACG | BamHI |
| | enhancer 2-BamHI/PstI | InvINH1-D5_F_BamHI | TTAAGGATCCGATTTTGGAAACACTCACAATCGT | BamHI | InvINH1-D6_R_PstI | ATTACTGCAGGCAATATTTGCTTTCCATATACGCAC | PstI |
| | enhancer 2ΔCArG-HindIII/XbaI | InvINH1-D5_F_HindIII | AATAAAGCTTTGATTTTGGAAACACTCACAATCGT | HindIII | InvINH1-D6M4_R_ XbaI | ATTATCTAGACTTTCCATATACGCACGTTCAAAGT | XbaI |
| | enhancer 2ΔCArG-XbaI/BamHI | InvINH1-D5_F | TTAATCTAGAGATTTTGGAAACACTCACAATCGT | XbaI | InvINH1-D6M4_R_ BamHI | ATTAGGATCCCTTTCCATATACGCACGTTCAAAGT | BamHI |
| Amplify enhancer 2 region for monomer or trimer construction | enhancer 2ΔCArG-BamHI/PstI | InvINH1-D5_F_BamHI | TTAAGGATCCGATTTTGGAAACACTCACAATCGT | BamHI | InvINH1-D6M4_R_ PstI | ATTACTGCAGCTTTCCATATACGCACGTTCAAAGT | PstI |
| | wildtype allele | SALK_022148 LP | GCATTTTCATTCTACAAACACCAATCC | n/a | SALK_137707 LP | AGACTGGGGAGGATAACCATG | n/a |
| | T-DNA allele for agl62-1 | LBb1.3 | ATTTTGCCGATTTCGGAAC | n/a | SALK_022148 LP | GCATTTTCATTCTACAAACACCAATCC | n/a |
| agl62 genotyping primers | T-DNA allele for agl62-2 | LBb1.3 | ATTTTGCCGATTTCGGAAC | n/a | SALK_137707 LP | AGACTGGGGAGGATAACCATG | n/a |
